# Shining light on the *Mary Rose*: Identifying chemical differences in human aging and handedness in the clavicles of sailors using Raman spectroscopy

**DOI:** 10.1371/journal.pone.0311717

**Published:** 2024-10-30

**Authors:** Sheona Isobel Shankland, Alexzandra Hildred, Adam Michael Taylor, Jemma Gillian Kerns

**Affiliations:** 1 Lancaster Medical School, Lancaster University, Lancaster, United Kingdom; 2 Mary Rose Museum, Portsmouth, United Kingdom; University of Szeged Institute of Biology: Szegedi Tudomanyegyetem Biologia Intezet, HUNGARY

## Abstract

The *Mary Rose*, for many years the flagship of the Tudor king, Henry VIII, sank during the battle of the Solent on the 19th July 1545. 437 years later, the remains of the hull and associated contents were recovered following a four-year excavation, all dated to a precise point in history. The assemblage is a valuable resource, as the environment preserved over 19,000 objects and the remains of a minimum of 179 crew members. This remarkable preservation allows for the crew of the *Mary Rose* to be studied holistically; their belongings, appearance, and even their health. Using Raman spectroscopy, this study investigated the clavicle bone chemistry of 12 men, aged 13–40, who died on the *Mary Rose*. Specifically looking at any changes with age or that could be linked to handedness. Results found that bone mineral increased with age and bone protein decreased. The mineral increase was found to be more substantial than the protein decrease. When the left and right side were considered, these findings maintained and were more pronounced in the right clavicle. This suggests that handedness influences clavicle bone chemistry; offering an important modern consideration for fracture risk. These results enhance our understanding of the lives of Tudor sailors, but also contribute to modern scientific investigation in the drive for a clearer understanding of changes in bone chemistry and potential links to aging related skeletal diseases such as osteoarthritis.

## Introduction

### The *Mary Rose*

**Fig 1:**
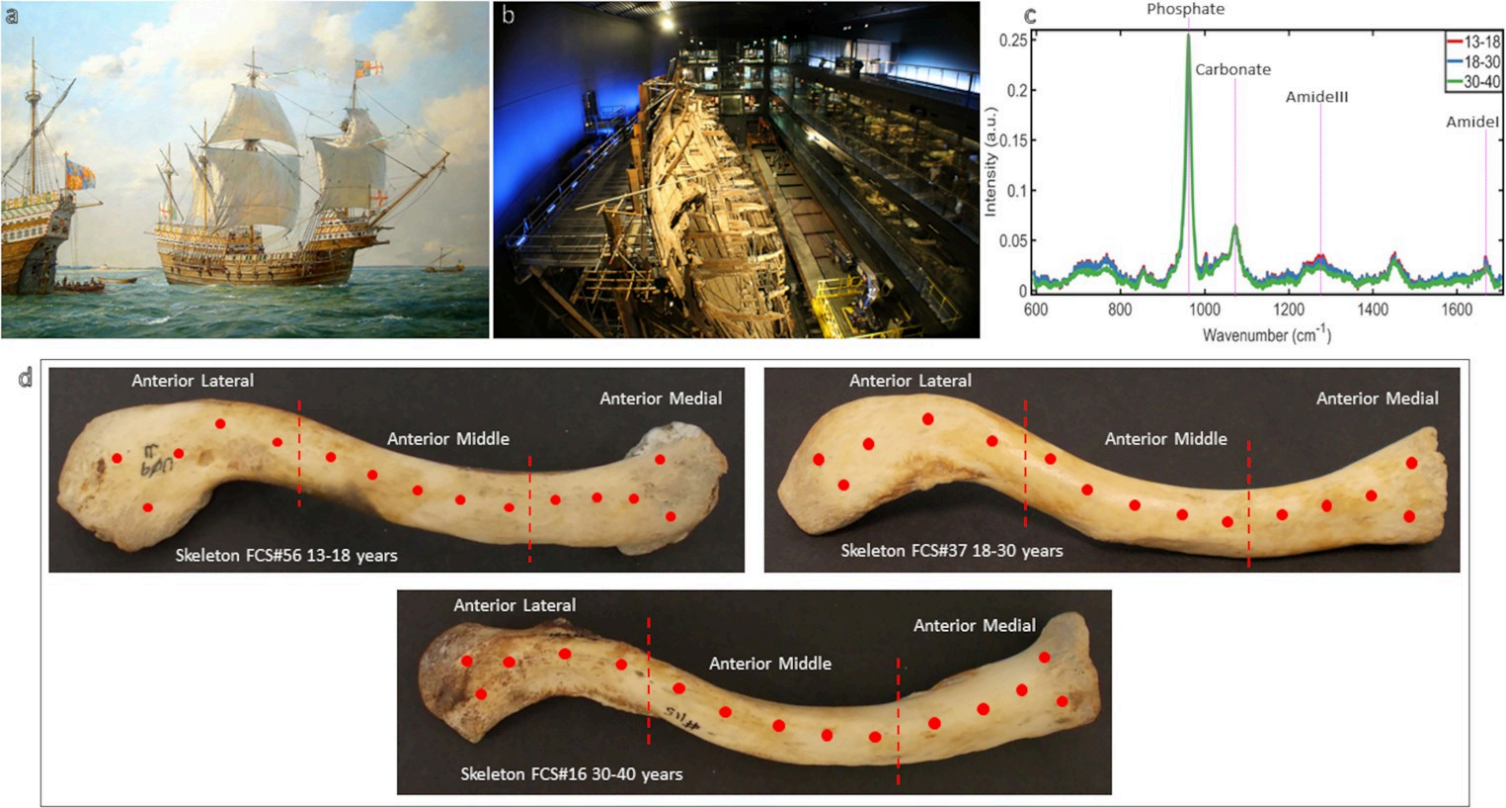
**(a)** The Mary Rose: Henry VIII’s Flagship, 1545. Painting by Geoff Hunt PPRSMA. Reprinted from The Mary Rose Trust under a CC BY license, with permission from The Mary Rose Trust, original copyright 2023 **(b)** Looking at the hull of the Mary Rose from the bow towards the stern within the Mary Rose Museum Portsmouth. Long glazed galleries opposite the hull contain some of the 25, 000 objects recovered from the excavation positioned opposite where they were found. Reprinted from The Mary Rose Trust under a CC BY license, with permission from The Mary Rose Trust, original copyright 2023 **(c)** Example spectra of the Mary Rose Clavicles taken from analyses of all measurements by age with annotations to illustrate the peak location for phosphate and carbonate, that relate to bone mineral, and amideI and amideIII, that relate to bone protein (collagen) **(d)** A schematic to illustrate the approximate location from which the measurements were taken; an example right clavicle from each age group.

The *Mary Rose* was one of the largest warships of the Tudor King, Henry VIII (r. 1509–1547) (Fig 1A and 1B) [1]. On 19^th^ July 1545, during the battle of the Solent off the south coast of England, the English fleet of ~80 vessels were launched towards ~225 invading French ships [2, 3]. Despite extensive research, the reason the *Mary Rose* sank remains a mystery. The most reliable account states the *Mary Rose* fired her guns from one side and turning to fire from the other side, she sank from water ingress through the open gun ports, claiming over 400 lives [4].

437 years later, on the 11th October 1982, the remaining hull of the *Mary Rose* was brought to the surface as the final part of a four-year underwater excavation which recovered thousands of objects and the partial remains of a minimum 179 individuals [2]. Thanks to the Solent tides, layers of soft sediment accumulated over the wreck creating an anaerobic environment; preserving artefacts and skeletal material in remarkable condition [5]. The high level of preservation of the skeletons provide rare insight into a precise point in human history and are also a valuable resource for modern scientific advancement.

Although most of the human remains were comingled, a number of individuals were isolated. From an assemblage of over 9000 individual bones, 98 individuals have been partially reconstructed (H. L. Matthews [Unpublished]). All remains were identified as men between ~10 and ~40years. From these individuals, this study aims to understand more about their lives and add to contemporary research on bone chemistry changes with age using Raman spectroscopy.

### Raman spectroscopy

Raman spectroscopy is a light-based technique that uses a monochromatic laser to excite the molecules of a sample. The vibrational changes that result from the energy exchange with the laser lead to an event referred to as Raman scattering [6]. Collecting this vibrational data allows the biochemical fingerprint of a sample to be established. This is an immensely valuable technique as it causes no damage, making it particularly important in archaeological research where samples are often priceless and irreplaceable. This technique has a diverse range of applications concerning bone and it has been able to identify a variety of mineral/function groups/pathogens in the skeleton [7–9]. It has advantages over other spectroscopic techniques as water is a relatively low Raman scatterer so the moisture of samples does not interfere with the results, and no sample preparation is needed, unlike IR spectroscopy that cannot be used on samples containing water. However, the Raman effect is weaker for some molecular bonds, therefore other spectroscopic techniques may be more appropriate, such as FTIR, which may have a higher sensitivity for low concentrations of certain chemical compositions.

### The clavicle

The clavicle, colloquially referred to as the collar bone, is a slim ‘S’ shaped bone that forms the anterior part of the shoulder girdle; the structure linking the upper limb to the torso. It is crucial in load transfer and motion of the upper limb, particularly when the arms are overhead as two thirds of shoulder elevation relies on the joint between the clavicle and the sternum (breastbone). It is one of the first bones to begin ossification (the laying down of mineral) in the human (approx. five weeks *in utero*), but is the last bone to fully fuse (approx. 22-25years [10]). This is due to a staggered ossification: the two primary centres fuse after five weeks of gestation at the middle of the clavicle, but there is a secondary ossification centre at the medial (sternal) end of the bone that appears ~17years and does not fully fuse with the rest of the bone until ~23years [11, 12]. This unique development allows for reliable age estimation in the *Mary Rose* crew, but also means that differences in bone chemistry may exist along the length of the bone relating to aging.

The surface of the clavicle is composed of cortical bone that is ridged where muscles attach. Muscles place bone under focused biomechanical stress and so their points of attachment increase in robusticity to compensate. This remodelling stress response exists across the entire skeleton in both the cortical shell and the spongy trabecular network within. The adaptive nature of bone makes it an ideal vessel to hold information on individual behaviours such as the manual life of a medieval sailor, but can also hold information about changes with disease, aging, or even handedness.

### Bone biology and mineralisation

The focus of this chemical analysis is on bone mineral chemistry and bone protein chemistry because these are the two largest components of bone. ~25% of bone is organic proteins, which are mainly collagen typeI (~90%), and ~65% is inorganic mineral, which is mainly hydroxyapatite (calcium phosphate). The remaining ~10% is bone cells and water [13, 14]. The bulk of bone mass is therefore located outside of bone cells in the extracellular matrix (ECM). The protein component gives bones some flexibility, and the mineral provides strength and rigidity. It is a delicate balance keeping the skeleton strong and supportive: not so rigid that force would cause fracturing, but not so flexible that the bone would bend. This elegant balance relies on signalling from bone cells to create the optimum skeletal design.

The collagen in the skeleton differs from elsewhere in the human body as it is mineralised [15]: the collagen fibrils, which are composed of a triple helix structure of α chains that are formed of amino acids, arrange themselves into a network that acts as a scaffold for hydroxyapatite crystals to attach [16]. The mineralisation mechanisms are not fully understood, but the process is known to be from calcium phosphate (apatite) interacting with hydroxyl ions in the ECM and undergoing nucleation to form clusters of a crystalline structure known as hydroxyapatite [17]. These crystals continue to proliferate and ions of carbonate are often substituted or included into the composition [15]. Carbonate substituted hydroxyapatite is plentiful and the biocompatibility is thought to be attributed to carbonate’s ability to reduce the apatite’s crystallinity, making it more soluble. This increased solubility improves bone turnover and remodelling [18, 19].

The hydroxyapatite-collagen association is the building block of successful bone tissue. Awareness of fluctuations in this relationship can enhance understanding of normal bone processes. However, there are many diseases and conditions that are affected by inefficiencies in this relationship; some are considered a result of aging, such as osteoarthritis and osteoporosis, but triggers for other conditions are not so readily suspected.

By using Raman spectroscopy to analyse the remains of the *Mary Rose* crew, this study aims to understand more about changes in bone chemistry, particularly with aging, so understanding of biological processes can be improved. The adaptive biology of bone leads to changes in chemistry and it is hypothesised that changes in phosphate, carbonate, and amine (the foundation of collagen) will be detected and be linked to aging, and possibly handedness. The *Mary Rose* crew are an ideal avenue for study as the nature of their preservation sets them apart from most archaeological human remains in that both the protein and mineral component of bone has been preserved by the anaerobic environment in which they were found. As such, these sailor’s remains are frequently used in research, allowing for reliable isotope analysis [1], investigation of bone diseases [5], taphonomy research [20], and assessment of injury and trauma [21], with a previous Raman spectroscopy study on *Mary Rose* samples determining the high quality of preservation by comparing the chemistry of Mary Rose specimens to the chemistry of modern samples with no significant spectral differences [5]. Given this established preservation, these remains provide an ideal opportunity for foundation research that is transferable to a modern clinical setting. This study will not only contribute to research on the variability of bone chemistry, but is important for learning more about our history, and proving archaeological remains can successfully be used to further modern medical research.

## Materials and methods

### Ethics statement

The specimens used in this study were provided by the Mary Rose Trust, Portsmouth, UK from the Mary Rose Museum, Portsmouth, UK. For specimen numbers please see S1 Table.

### Materials

10 pairs of clavicles from 10 individuals, one single left clavicle, and one single right clavicle from different individuals were provided by The Mary Rose Trust (S1 Table). The individuals to whom these bones belong were 13-40years and were divided into three age groups: 13–18 (youngest group), 18–30 (middle group), and 30–40 (oldest group). These ages were determined by Mary Rose Trust osteologists prior to this study. All remains were found within the hull during excavation and represent members of the crew who were aboard when the ship sank.

No cleaning or preparation was required on these samples and they were received and returned in the same condition.

### Data collection

A Renishaw inVia (Renishaw plc. Gloucestershire, UK) Raman microscope (x20 long objective lens) with a 785nm laser of 0.2mm spot-size was used in this study. The instrument was calibrated using silicon and polystyrene. Spectra were acquired using 50% (~1mW at sample) laser power for 60 accumulations at 2 seconds. The spectral range was 1704 to 594cm^-1^ and 336 spectra were collected along the anterior surface of the clavicle.

### Measurements

The clavicle was placed on the instrument stage on its long axis on its posterior side. 15 measurements were taken along the anterior surface at approx.10mm intervals, ensuring no overlap with previous measurements. Adjustments were made to avoid areas with iron staining. These 15 measurements were divided into three sections of five measurements each, dividing the clavicle into three parts: lateral, middle, and medial (Fig 1D). Measurement locations were selected due to their presence on every sample and the lack of direct muscle attachment or articular surface, to limit the influence of extraneous variables. All measurements were taken from intact cortical bone.

### Data analysis

#### Pre-processing

Pre-processing and analyses were carried out on the full spectral range of the raw data using MATLABR2017a with an ‘irootlab’ coding plugin [22–24]. All data were baseline corrected using a polynomial of 7 before vector normalisation. These processed data were then analysed using Principal Components Analysis-Linear Discriminant Analysis (PCA-LDA) to investigate changes in clavicle chemistry with aging, considering side, region, and measurement location.

#### Analysis

A combined PCA-LDA approach was used to analyse the chemical variance of these samples. PCA reduces the dimensionality of data by identifying and retaining the most important features in the dataset (the principal components). This results in simplified data that preserves as much variance as possible. LDA then finds linear combinations of the features that best separate the different classes in the dataset, maximising the discrimination between and within classes. The output were scores plots and accompanying loadings. The scores plots allow for observation of trends between classes and the loadings allow discernment of the chemical responsible for trends, should they be found. This analysis allows subtle differences to become clearer and any changes to be corelated with aging and handedness [25].

Initial ratios of 960:1450 cm^-1^ were calculated to determine the collagen preservation from the average spectrum of the outer surface of these samples [26, 27]. Intra mineral (phosphate (~960cm^-1^) to carbonate (~1070cm^-1^)) ratios and mineral to protein (phosphate (~960cm^-1^) to amideIII (~1270cm^-1^)) ratios were also determined. Ratios were calculated from the values of the true peak heights, which were determined from the difference between the peak height and the baseline measurements. The relative maturity of the mineral crystals was calculated using the full width at half the height of the phosphate peak (~960cm^-1^) [8].

## Results

All measurements were taken from the cortical bone of the clavicle, so were inherently very similar in their composition (Fig 1C), however, the changes in chemical minutiae with age can be investigated.

Visualising chemical trends with aging using 2D scores plots is useful to illustrate the relationships of the chemical changes. The chemical trends demonstrated by our analyses showed remarkable consistency as demonstrated by the loadings. Due to this consistency, specific chemical contribution to the scores plot trends were not always identifiable.

Collagen quality in these samples was calculated using the ratio of phosphate (~960cm^-1^) to 1450cm^-1^ and was found to be 8.01, which indicates that the protein is well-preserved in these samples [27].

### Analysed by age (Fig 2)

**Fig 2:**
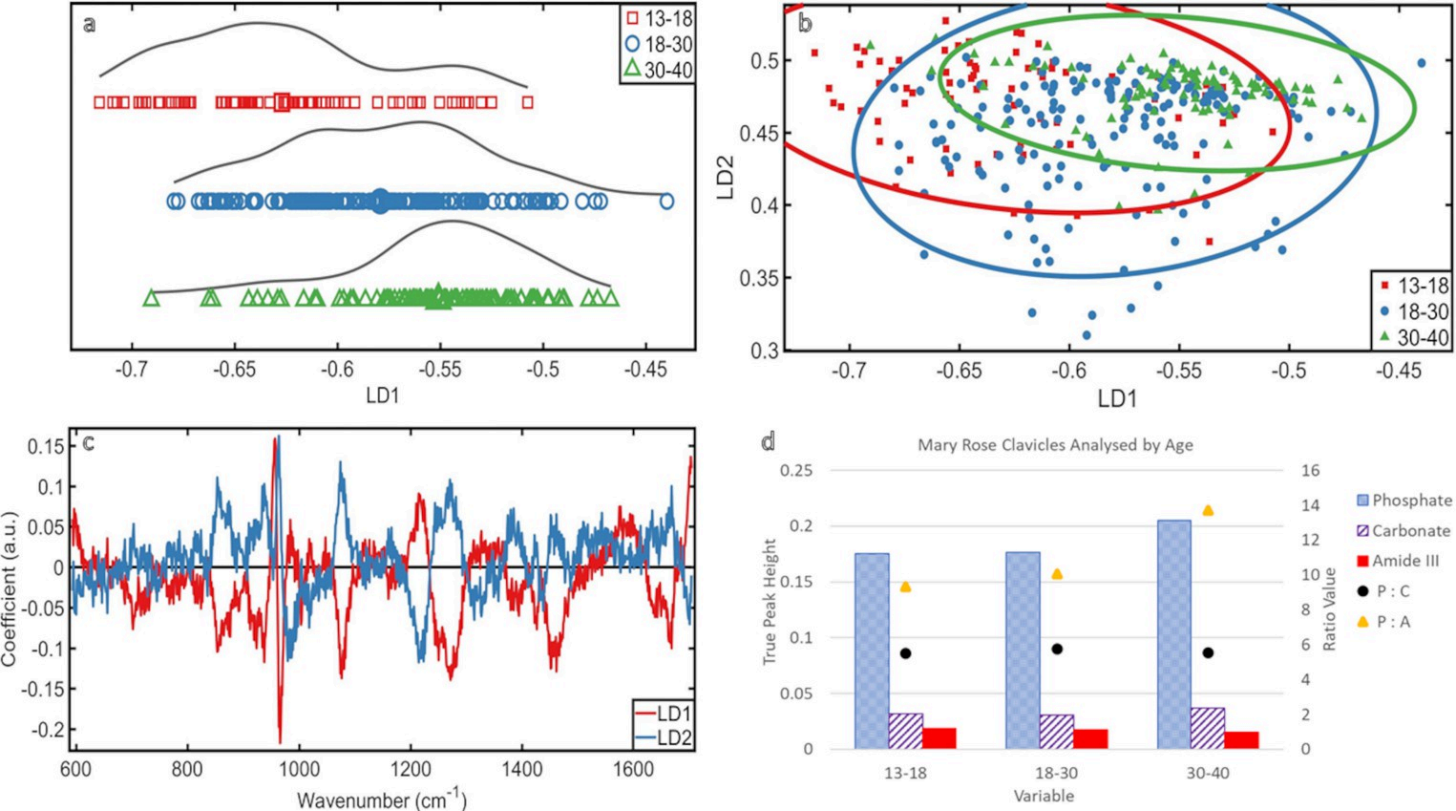
Analysed by Age: **(a)** PCA-LDA 1D scores plot **(b)** PCA-LDA 2D scores plot **(c)** PCA-LDA loadings plot **(d)** ratio plot.

When all measurements were analysed by the age of the individual, the age groups separated left to right (Fig 2A). There was substantial overlap (which is expected as samples have age ranges), but separate averages illustrated a change in overall clavicle chemistry with aging.

When viewed in a 2D scores plot (Fig 2B), the degree of overlap meant there was no clear distinction. However, the middle group had wider distribution over LD2, and the younger and older groups had a narrower distribution.

From the loadings (Fig 2C), it was determined that changes over both LDs are predominantly due to phosphate (~960cm^-1^). The chemical intensity in the spectra showed the oldest group had the highest relative amount of phosphate and the middle and younger groups were similar and had less. This indicated a more substantial phosphate change in older skeletons, which was found to be an increase in phosphate intensity (Fig 2D). Changes in carbonate (~1070cm^-1^) were also prominent across both LDs (Fig 2C) with the oldest group demonstrating the highest intensity. Followed by the youngest group, then the middle group (Fig 2D).

Less strongly, but still present, were changes in amideIII (~1270cm^-1^) and in proline (~850cm^-1^). The intensity of the proteins decreased with aging; from youngest to middle to oldest (Fig 2D).

The phosphate to carbonate mineral ratio (P:C) did not show a substantial change with aging, though the pattern suggested an increase from the younger to middle group, then a decrease from the middle to older group. The ratio of phosphate (the more prevalent mineral) to amideIII (the most prevalent protein feature detected) (P:A) showed an increase with aging, but this ratio increase was greater between the middle and older group.

The most mature mineral was found in the older group, and the least mature in the middle group (S2 Table), however, these changes were not substantial.

In the clavicle as a whole, mineral increased with age (both phosphate and carbonate) whilst protein (amideIII) decreased. As the analyses demonstrated a stronger influence in phosphate and carbonate variation, the increase in mineral was more substantial than the decrease in protein.

### Analysed by age and side (Fig 3)

**Fig 3:**
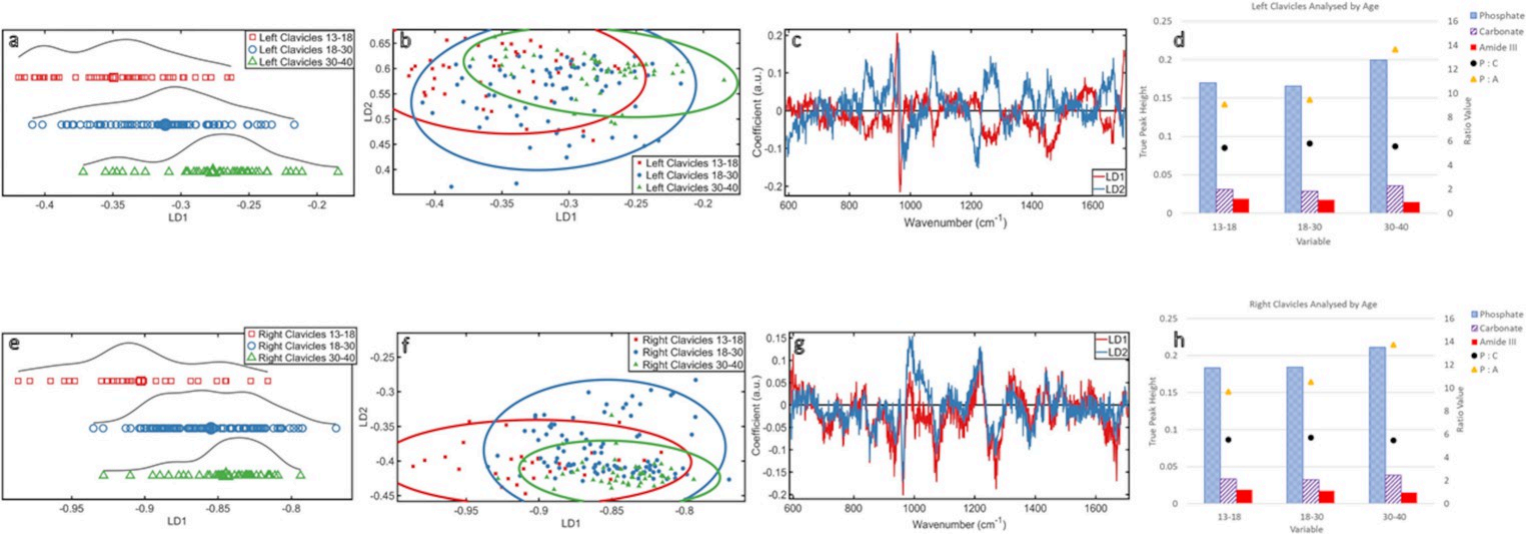
Analysed by Age and Side: **(a)** ByAge_LeftClavicles PCA-LDA 1D scores plot **(b)** ByAge_LeftClavicles PCA-LDA 2D scores plot **(c)** ByAge_LeftClavicles PCA-LDA loadings plot **(d)** ByAge_LeftClavicles ratio plot **(e)** ByAge_RightClavicles PCA-LDA 1D scores plot **(f)** ByAge_RightClavicles PCA-LDA 2D scores plot **(g)** ByAge_RightClavicles PCA-LDA loadings plot **(h)** ByAge_RightClavicles ratio plot.

The aging pattern was mirrored in both sides in the 1D scores plot (Fig 3A and 3E). The younger group was more separate from the middle and older groups than they were from each other and this pattern was stronger in the right clavicles. The distribution of the left 13–18 data showed a bimodal distribution, meaning this age group can be partially separated into two chemical profiles. The right clavicles showed a more normal distribution with a less distinct split. The 18–30 data for both sides was more normally distributed with a wider spread, indicating more heterogeneity in this group. Specific chemical changes were therefore less distinct. The distribution of the 30–40 data had a distinct grouping to the right of the plot, with some in-class separation on both sides, this was stronger on the left. The left clavicles presented a stronger chemical separation within age groups.

In the 2D scores plot (Fig 3B and 3F), there was clear overlap amongst all groups in both sides, but both middle groups showed the most spread over LD2. However, the right clavicles of the older group fully reside within the middle group spread across both LDs. The spread of the younger group over LD1 was more defined by age in the right clavicle.

The loadings (Fig 3C and 3G) showed the changes in both clavicles over both LDs were attributed to phosphate (~960cm^-1^) and carbonate (~1070cm^-1^). The chemical intensity in both sides showed the oldest group had the highest relative amount of phosphate (Fig 3D and 3H), the youngest and middle group were lower and similar in their intensity. The oldest group had the highest intensity of carbonate (~1070cm^-1^), in both clavicles, followed by the youngest then the middle group, although they were similar (Fig 3D and 3H).

Though weaker than mineral values, substantial changes with age were detected relating to amideIII (~1270cm^-1^) (Fig 3C and 3G). For both sides, the highest intensity was in the youngest group, then the middle group, then the oldest (Fig 3D and 3H). The loadings also showed changes in amideI (~1670cm^-1^) for the right clavicle (Fig 3G) and the intensity decreased with aging. 1450cm^-1^ also contributed to LD1 trends in the left clavicle (Fig 3C), this CH_2_ band is indicative of the protein quality (potentially reflecting the proportion of type1 collagen present).

The change in P:C with aging was the same on both sides (Fig 3D and 3H). This demonstrated an increase from the younger to middle group, then a decrease from middle to older group, but these changes were minor. The P: A increased with age on both sides. This ratio increase was stronger from middle to older group than in younger samples. There was no clear difference between sides.

The most mature mineral was found in the right clavicles of the youngest group and the least mature in the left clavicles of the middle group, but the differences were slight (S2 Table).

Chemical changes with age were confirmed over LD1. There were differences in data distribution between sides, but lack of distinction suggested the changes between sides are minimal. The changes with age shown in Fig 2 are maintained when the clavicles are separated into left and rights, but these changes appear stronger in the right clavicles.

### Analysis by age medial vs. Lateral measurements (Fig 4)

**Fig 4:**
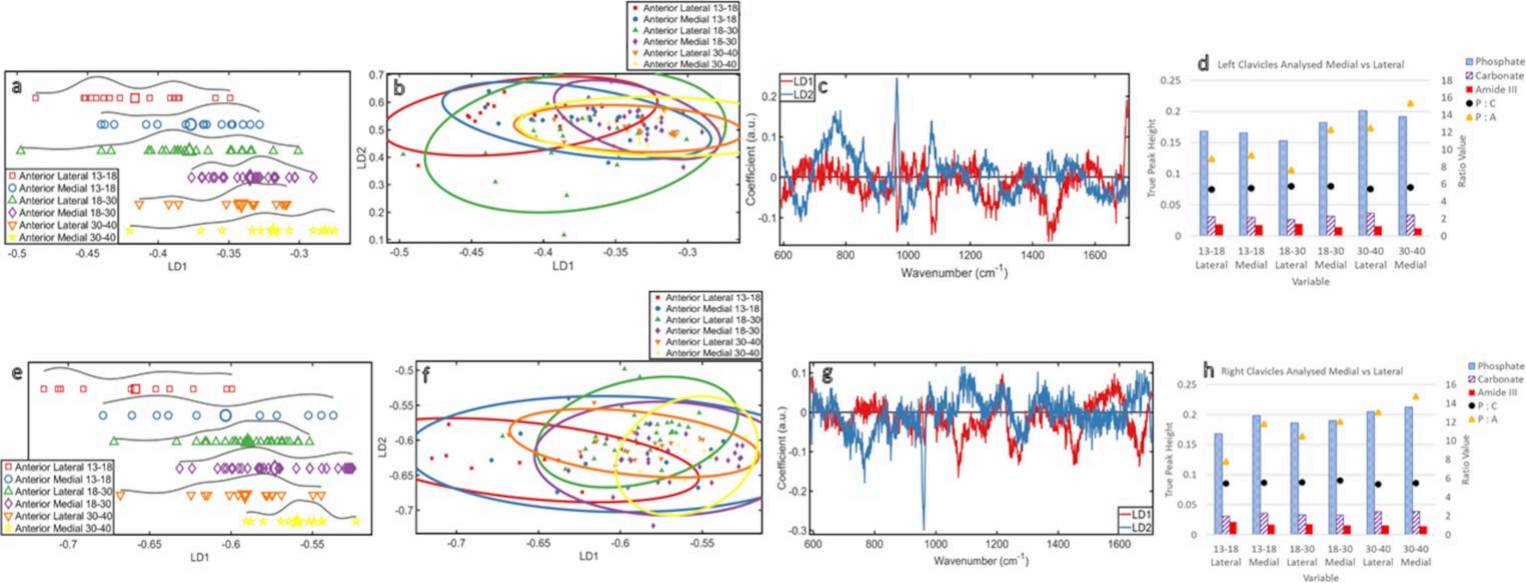
Analysed by Age Medial vs. Lateral: **(a)** ByAge_MedvsLat_LeftClavicles PCA-LDA 1D scores plot **(b)** ByAge_MedvsLat_LeftClavicles PCA-LDA 2D scores plot **(c)** ByAge_MedvsLat_LeftClavicles PCA-LDA loadings plot **(d)** ByAge_MedvsLat_LeftClavicles ratio plot **(e)** ByAge_MedvsLat_RightClavicles PCA-LDA 1D scores plot **(f)** ByAge_MedvsLat_RightClavicles PCA-LDA 2D scores plot **(g)** ByAge_MedvsLat_RightClavicles PCA-LDA loadings plot **(h)** ByAge_MedvsLat_RightClavicles ratio plot.

When the lateral and medial measurements were isolated on each side and viewed as a 1D scores plot (Fig 4A and 4E), the medial measurements showed a stronger chemical change with aging than the lateral ones in every age group. There was also an overall increased homogeneity with aging. However, while the pattern in the left and right was similar, changes in chemistry within the younger group were more pronounced between sides than in the middle and older groups.

In the 2D scores plots (Fig 4B and 4F), the lateral measurements of the middle group’s left clavicles show the most spread over LD2; all other groups directly overlap. The right clavicles of the youngest group show a wider spread over LD1 than LD2 for the medial and lateral measurements. The other two age groups are more evenly distributed between LD1 and LD2. This distinction was not visible when the data were only separated by age and side.

The loadings (Fig 4C and 4G) show the changes with age in both the left and right clavicles that are apparent over LD1 can be attributed to carbonate (~1070cm^-1^) and (~1450cm^-1^). The carbonate intensity was highest in the oldest group (lateral then medial) for both sides. The other groups had too much overlap to determine a pattern. Carbonate was also identified as a contributor to LD2 changes in the left clavicles, but trends in Fig 4B were not distinct enough to contribute to a conclusion about the relationship between carbonate and aging. 1450cm^-1^ also contributed to LD1 trends, this CH_2_ band is indicative of the protein quality and was highest in the youngest clavicles (lateral then medial) on both sides. The other groups were consistently lower in intensity and decreased with aging from lateral to medial, but were only distinct in the left clavicles. No trend or pattern was observed in the right clavicles.

The influence of phosphate (~960cm^-1^) in the loadings is stronger in LD2 for both sides, so any potential trends (Fig 4B and 4F) would likely be a result of changes in phosphate. Phosphate changes were only contributing over LD1 in the left clavicles. The overall phosphate intensity in the left clavicles was highest in the older group (lateral then medial measurements). The overall phosphate intensity in the right clavicles was highest in the older group (but medial then lateral measurements). There was no clear pattern with aging in left and right clavicles for younger and middle groups when the data were separated by lateral and medial measurements.

Over LD1, there were changes to wavenumbers relating to amideIII (~1270cm^-1^) with aging for the medial and lateral sections of the right clavicles. The intensity of amideIII was highest in the youngest group (lateral then medial); the remaining groups overlapped, except for the older group medial measurements, which had the lowest intensity. LD1 also showed changes at amideI (~1670cm^-1^) for the right clavicle. The intensity was highest in the youngest group lateral measurements, then the middle group (lateral then medial), then the youngest medial measurements, then the older group (lateral then medial).

The P:C (Fig 4D and 4H) did not show notable changes with age between ends of the clavicles or between sides. The potential pattern observed in previous analyses was maintained, but the isolation and comparison of the medial and lateral measurements contributed new information that the medial measurements could have a higher mineral ratio than their lateral counterparts for both sides and all age groups. The P:A increased with age and was consistently higher in the medial measurements than the lateral of each age group, for both sides. This medial to lateral difference was most pronounced in the middle group left clavicles and the youngest group right clavicles. Overall, P:A increased with age, but the lateral measurements from the middle group were lower than the medial measurements of the youngest group for both sides.

The most mature mineral was found in the right medial measurements of the youngest group and the least mature was found in the left lateral measurements of the middle group. However, the differences in crystal maturity values were minor. (S2 Table).

This further analysis, separating the measurements taken from either end of the clavicles, showed that the pattern of increased mineralisation with age found in earlier results is consistent and the P:A is consistently higher in the medial measurements than the lateral. This pattern comes from the medial measurements having consistently more mineral and lower protein than their lateral counterparts. Fluctuations in the proportions of the minerals continues to be negligible.

### Analysis by age and side: Lateral vs. Middle vs. Medial (Fig 5)

**Fig 5:**
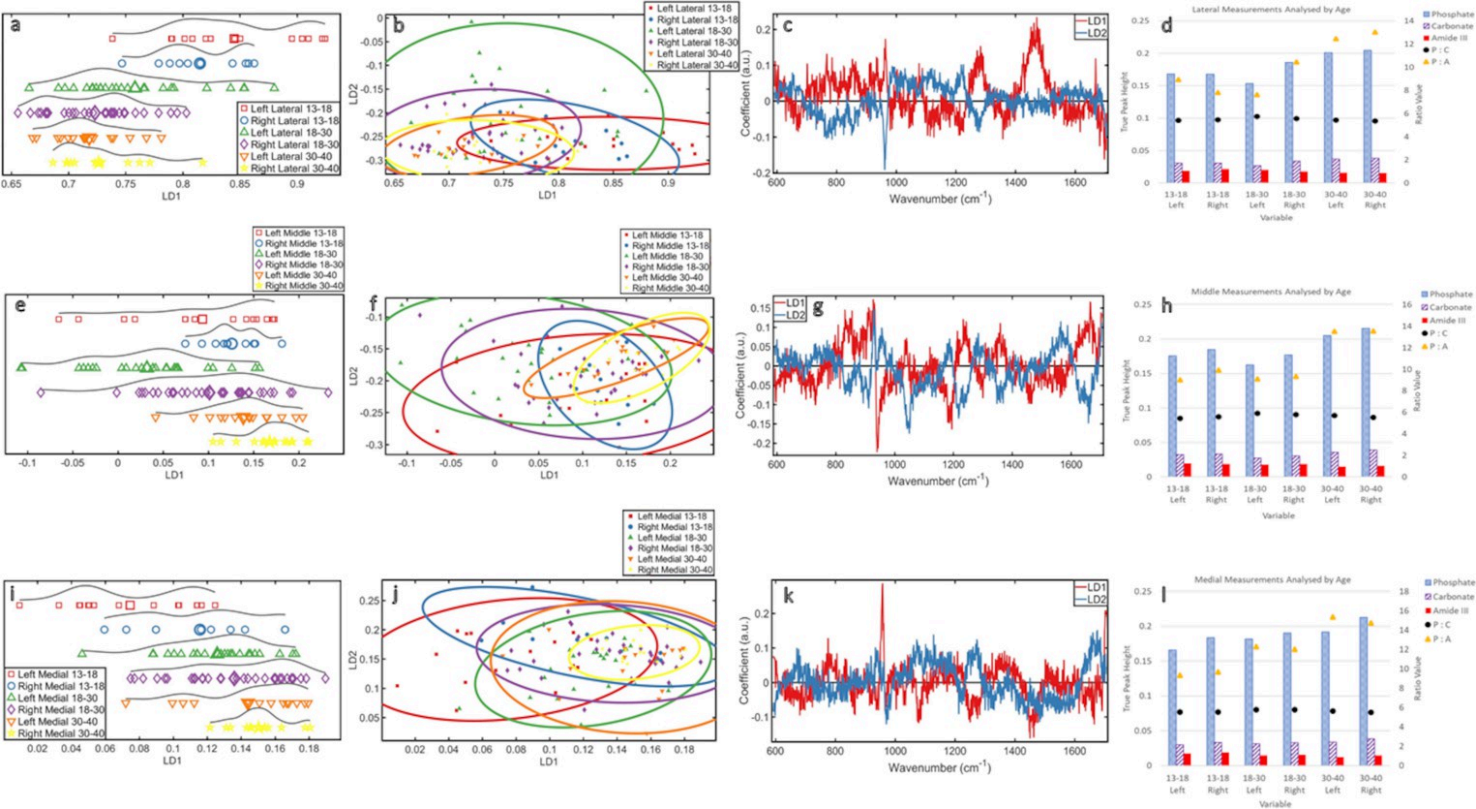
Analysed by Age Lateral vs. Middle vs. Medial: **(a)** ByAge_LeftvsRight_Lateral PCA-LDA 1D scores plot **(b)** ByAge_LeftvsRight_Lateral PCA-LDA 2D scores plot **(c)** ByAge_LeftvsRight_Lateral PCA-LDA loadings plot **(d)** ByAge_LeftvsRight_Lateral ratio plot **(e)** ByAge_LeftvsRight_Middle PCA-LDA 2D scores plot **(f)** ByAge_LeftvsRight_Middle PCA-LDA 2D scores plot **(g)** ByAge_LeftvsRight_Middle PCA-LDA loadings plot **(h)** ByAge_LeftvsRight_Middle ratio plot **(i)** ByAge_LeftvsRight_Medial PCA-LDA 2D scores plot **(j)** ByAge_LeftvsRight_Medial PCA-LDA 2D scores plot **(k)** ByAge_LeftvsRight_Medial PCA-LDA loadings plot **(l)** ByAge_LeftvsRight_Medial ratio plot.

In a 1D scores plot, chemical changes caused the youngest group to be separated from the middle and older groups in the lateral measurements, with more heterogeneity in the left clavicles (Fig 5A). The middle and older group showed a similar distribution and did not show distinct changes between sides, though there was an increase in homogeneity with age. Changes in the middle measurements did not possess an age or side related pattern, but again there was more heterogeneity in the left clavicles for the youngest group when compared to the right (Fig 5E). Again, there was an increase in homogeneity with age when considering the middle and older groups. The left measurements of the middle group also appeared to align more closely with the youngest group’s distribution, than with its right counterpart. There was a clearer pattern in the medial measurements which showed a distribution with age seen in previous analyses as well as an increase in homogeneity with each age group (Fig 5I). The right measurements were consistently separated in the scores plot from the left measurements, indicating more similarity with the likely non-dominant side of the age group above. Again, there was an increase in homogeneity with age, however, for the medial measurements this was seen through all three age groups, instead of only the older two as seen in the lateral and middle analyses. Across all LD1 plots there was no consistent pattern for each group in any region, but every region showed an increase in chemical homogeneity with age.

The 2D scores plot showed changes in the lateral measurements of the left side of the middle group were the only considerable changes over LD2 (Fig 5B). The 2D scores plot for middle measurements illustrated that the increased homogeneity with age can be well observed over LD2 (Fig 5F). The right side of the older group was the most homogeneous in all three measurement locations, and lay within the distribution of the other groups. Overall, there was too much overlap in the LD2 distribution to relate any LD2 chemical changes to a trend in bone chemistry with aging, but an increased in homogeneity was found in all three 2D scores plots (Fig 5A, 5F and 5J).

The loadings showed changes across the lateral measurements with age were due to phosphate (~960cm^-1^) over both LDs, and amideIII (~1250cm^-1^) and 1450cm^-1^ over LD1 (Fig 5C). Phosphate (~960cm^-1^) intensity was highest in the oldest group (right then left), then the right measurements of the middle group, then the youngest group (left then right) and finally the left measurements of the middle group. AmideIII (~1250cm^-1^) intensity was highest in the youngest group (right then left), then the middle group (left then right), then the oldest group (right then left). 1450cm^-1^ intensity was highest in the youngest group (left then right), the middle group and the right clavicles of the older group were all equal in intensity, the lowest intensity was the left side of the oldest group.

Changes across the middle measurements with age were due to phosphate (~960cm^-1^), amideIII (~1270cm^-1^) and amideI (~1670cm^-1^) over both LDs and carbonate (~1070cm^-1^) over LD2 (Fig 5G). Phosphate (~960cm^-1^) was highest in the oldest group (right then left), then right measurements for the youngest group, then an equal intensity for left measurements of the youngest group and the right measurements of the middle age group. The lowest intensity was in the left measurements for the middle group. AmideI (~1670cm^-1^) presented with a high degree of overlap, but the lowest intensity was the oldest group (left then right). AmideIII (~1270cm^-1^) intensity was equally highest in the left clavicles of the youngest and middle group, then the right middle group, then the right youngest group. The oldest group had the lowest intensity (left then right). There was too much overlap in carbonate intensity to determine a pattern.

Changes across the medial measurements with age were due to changes in phosphate (~960cm^-1^) and ~1450cm^-1^ over LD1 (Fig 5K). Phosphate intensity was highest in the right measurements of the oldest group, then the rights of the youngest group. Next were the left measurements of the oldest group, right of the middle group, the left of the middle group and finally the left of the youngest group. 1450cm^-1^ intensity was highest in the left clavicles of the youngest group, then equally in the left clavicles of the middle and oldest group, then the right middle group and finally the right clavicles of the oldest group.

When the data were viewed as ratios, there were no major differences found between minerals within or between measurement areas (Fig 5D, 5H and 5L). There were indications of a higher P:C in the middle measurements as was found in all earlier analyses, but it was negligible. P:A ratios were consistently highest in the older group of all three measurement areas as expected from earlier analyses, but there was no side related pattern apparent with aging and no other clear pattern in these ratios were observed in this analysis.

The least mature mineral crystals were found in the left lateral measurements on the middle group, and the most mature were found in the right medial measurements of the oldest group, but these findings were minor. (S2 Table).

In both scores plots (Fig 5A, 5B, 5E, 5F, 5I and 5J), the older groups were consistently more homogeneous for all areas which suggested that the chemical variation in each measured region decreases with age. While the loadings indicated that the mineral and protein are causing these changes in distribution in a way that potentially correlates with other analyses in this study, the lack of clear pattern means that conclusions cannot be formed on left and right-side differences in measured regions.

### Analysed by age and side: Measurement location along length of clavicle (Fig 6)

**Fig 6:**
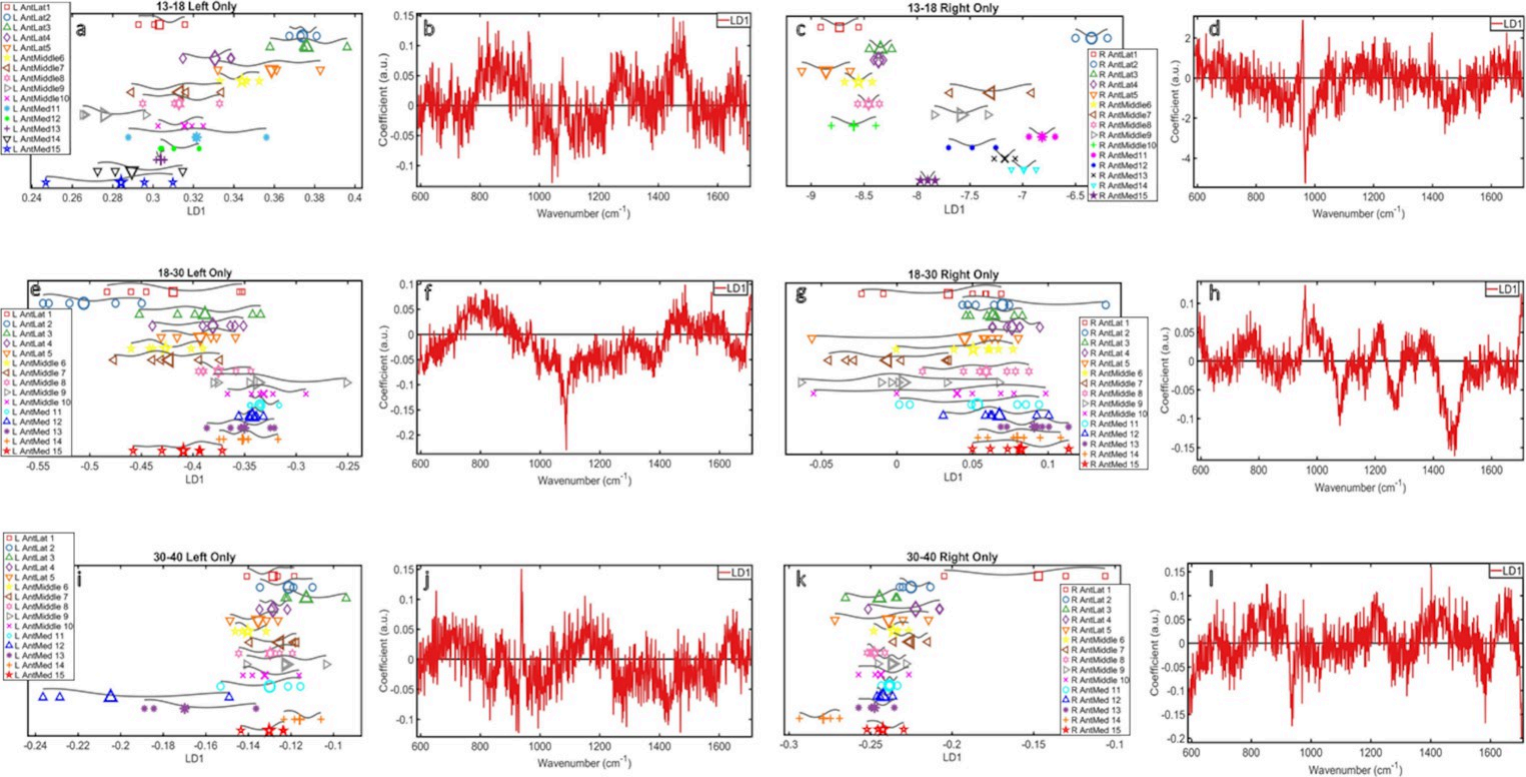
Measurements along the clavicles analysed by age and side: **(a)** 13–18 Left Clavicles 1D scores plot **(b)** 13–18 Left Clavicles loadings plot **(c)** 13–18 Right Clavicles 1D scores plot **(d)** 13–18 Right Clavicles loadings plot **(e)** 18–30 Left Clavicles 1D scores plot **(f)** 18–30 Left Clavicles loadings plot **(g)** 18–30 Right Clavicles 1D scores plot **(h)** 18–30 Right Clavicles loadings plot **(i)** 30–40 Left Clavicles 1D scores plot **(j)** 30–40 Left Clavicles loadings plot **(k)** 30–40 Right Clavicles 1D scores plot **(l)** 30–40 Right Clavicles loadings plot.

**13–18.** Along the length of the left clavicles, there was a pattern of distribution over LD1 from lateral to medial, with the exception of the primary lateral measurement (Fig 6A). The loadings indicated that any patterns were a result of changes in carbonate (~1070cm^-1^) and ~1450cm^-1^ (Fig 6B). At ~1070cm^-1^ all measurements appeared equal in intensity. Along the length of the right clavicles there was no clear trend in distribution, but the data alternated over a mid-point along the clavicle length. The loadings indicated that the strongest chemical changes detected were in phosphate (~960cm^-1^) (Fig 6D), but these changes could not be attributed to a pattern along the clavicle length.

**18–30.** There was a trend of increasing LD1 value towards the middle of the left clavicles from the lateral aspect and then a decreasing LD1 value from the middle to the most medial measurement (Fig 6E). The loadings indicated any patterns were a result of changes in carbonate (~1070cm^-1^) (Fig 6F), but there was very little differentiation in intensity between groups. Along the length of the right clavicle there was no clear pattern, but the distribution of the data for each group was more heterogeneous in the middle than the lateral and medial ends (Fig 6G). The loadings indicated that the strongest chemical changes were in phosphate (~960cm^-1^), carbonate (~1070cm^-1^), and 1450cm^-1^, but there was no trend with which to associate these changes as the intensities overlapped considerably (Fig 6H).

**30–40.** From lateral to medial across the left clavicles, the LD1 did not show a pattern of change across the length (Fig 6I). The only differences identified in the scores plot related to two areas in the medial clavicle. The loadings suggest this could be due to changes in phosphate (~960cm^-1^) (Fig 6J), but due to overlap no pattern was observed, potentially indicating chemical consistency along the clavicle length.

From lateral to medial across the right clavicles, the LD1 did not show a pattern of change across the length, the only group showing change was the primary lateral measurement (Fig 6K). There was little heterogeneity along the length of the right clavicles and did not possess substantially different chemical compositions as each group resides on a similar LD1 value. The loadings indicated any patterns were a result of phosphate (~960cm^-1^) (Fig 6L). At ~960cm^-1^ there was very little differentiation in intensities, the only measurement showing any separation was the first lateral measurement which was lower than all other measurements. This is reflected in the LD1 plot.

When examining the ratios (S1 Fig), P:C held steady along the length at a consistent value across all groups. P:A did not present with a pattern between sides or ages.

The mineral crystal maturity (S2 Table) also did not present with a clear pattern along the clavicle lengths.

### Crystal maturity

While mineral crystal maturity was investigated, all analyses indicated very little change (S2 Table). As no major, or potential, trends were detected, contribution of crystal maturity was not considered further.

## Discussion

### Analysis by age

Changes in bone chemistry with aging were detected (Fig 2): mineral increases with age and protein decreases; mineral increase was more substantial than protein decrease. This could be due to collagen still being formed in the bones of the younger individuals while it is depleting in older individuals [28, 29]. The age range nature of the data classification potentially affects how the distributions relate to chemical changes. Also, lived age does not directly correspond to biological age. There could be substantial chemical influences causing changes in the younger and older groups as they show clusters in the scores plots, which could be showing the gradual transition of bone chemistry changes with age or chemical stability within the age group as was found in the middle group.

### Analysis by age and side

The data distributions in Fig 3 indicate that, while the mineral and protein changes found in Fig 2 are appearing in both sides, they are more defined in the right clavicles. A previous study found that non-dominant side clavicles possessed greater mineral mass than the dominant side [30]. As samples in this study come from a medieval population where left handedness was strongly discouraged due to its associations with witchcraft combined with the generally higher incidence of right handedness in humans [31], fluctuations in left or right dominance was discarded from this study and right sided preference was assumed. Considering these facts alongside previous findings, it would be expected that the left clavicles would possess a greater mineral mass than the right [30]. The findings in this study reveal changes with age are more defined in the right side. Assuming right side dominance, this could mean more defined bone chemistry due to remodelling from repeated activity on board a ship, instead of relying on the individual nature of biological aging, which would be less defined [32, 33]. When the pattern of bone mineral intensity was investigated (Fig 3D and 3H) the right clavicles have relatively higher bone mineral (combined and isolated phosphate/carbonate) than the left in all age groups, even in the middle group which most directly compares to the cohort of the previous study [30]. This study has detected that the right clavicles are more highly mineralised than the left in all three age groups in addition to the finding that bone mineral increases with aging. This contradicts previous findings [30]. This could be a result of handedness being a more considered issue in modern populations [32] and/or the labour-intensive lifestyles of the sailors whose clavicles were analysed in this study. It is also worth considering that the previous study used densitometry; a scan that only assesses bone mineral density based on macroscopic data; a methodology used by many studies investigating bone mineral [34, 35]. The use of Raman spectroscopy allows for a more detailed assessment of bone mineral, so is likely giving a more comprehensive assessment of bone mineral quantity than macroscopic assessment methods. This finding suggests that Raman spectroscopy could be a more effective diagnostic tool than is currently used in clinical assessments and research into clavicle bone mineral density/quantity.

With a focus on chemical changes with aging, the youngest age group for the left samples shows a split. As this age group is composed of individuals aged of 13–18 it is likely that this split is a result of the more extreme changes that happen in early adolescence as the skeleton is still developing, compared to late adolescence where the skeleton is classed as an adult. This split is thought to be more pronounced in the left as the natural changes of development are clearer without the influence of activity related remodelling on the right dominant side. This is mirrored in the older group, but for the reasons of degradation rather than development. The more even distribution observed in the middle group’s plots is thought to be a result of both the transitional nature of the age group coupled with this middle age range encapsulating the age considered to be the time of most biological stability in bone [18]. This stability is reflected in the 2D scores plots (Fig 3B and 3F) as the middle group has a more even distribution over LD1 and LD2 than the younger and older groups. However, there is no notable change to bone chemistry when the data is separated by side that can be isolated as the cause. The changes in bone chemistry show relatively higher phosphate (~960cm^-1^) in the older clavicles on both sides, and the younger and middle clavicles are more similar to each other with less phosphate. Both sides are also influenced by changes in carbonate (~1070cm^-1^), again with the highest intensity in the older group. Though this was stronger on the right there was no clear chemical change relating to side.

The presence of the CH_2_ (~1450cm^-1^) band in this analysis is indicative of the integrity of the organic components in bone, particularly collagen as it is the most abundant organic component [26]. As these samples had a strong Raman signal at 1450cm^-1^ and a ratio value of 8.01 for the 960:1450cm^-1^, this indicated that 100% of the collagen at the outer surface of the samples is classified as well preserved [27]; intact collagen which can be studied [36], particularly with the benchtop Raman microscope used in this study over a handheld device. Handheld devices, despite advances in their spectral sensitivity and resolution, do not currently match the output quality of a benchtop instrument. Their noisier spectra make it difficult to distinguish smaller peaks, especially in archaeological studies where these devices are often used in the field when experimental conditions are less desirable [27]. The absence of a CH_2_ band would indicate that the protein was too degraded to study [8, 27, 36]. AmideIII decreased with age on both sides, but this was weaker on the left. Chemical changes with age for the right clavicles also affected amideI; this protein’s presence also decreased with age. The strength of these changes could relate to increased remodelling with right handedness, but they are not chemically distinct enough to be certain.

The ratios of these chemical changes show there were no strong changes in the type of mineral with age between or within sides. P:A did not show any changes that could be linked to handedness, but did show an increase with aging in the same pattern that was found when the data were analysed as a whole *i*.*e*. were not separated by side.

### Analysis by age: Medial vs. Lateral measurements

When the left and right clavicles of the three age groups are also separated by their most medial and lateral measurements (Fig 4), the chemical changes in the medial measurements were stronger than in the lateral measurements of the same side and age group. This trend is likely due to differences in bone remodelling between clavicle ends. Each end of the clavicle has different biomechanical demands which leads to differences in bone chemistry. A reduced need for bone remodelling would mean less chemical change, which relates to the lateral measurements. This supports findings by a previous study which showed that the role of the lateral clavicle means it may have a reduced need for bone remodelling [37]. In the present study, these changes over LD1 were associated with carbonate for both sides and as carbonate increases the solubility of bone mineral to enable bone turnover [18] it follows that there was more carbonate change detected in the medial measurements than the lateral ones. This LD1 distribution pattern could also be linked to the late ossification of the medial end of the clavicle causing more chemical changes, particularly as there is an increase in homogeneity with age in the medial measurements from younger to middle group in both sides, which could show the fusion of the later medial ossification area with the rest of the clavicle [12]. The separation of the medial and lateral data indicated a pattern of increasing homogeneity with aging, but most heterogeneity was found in the right clavicles of the youngest group; likely linked to both development and handedness. There is less chemical distinction in developing bones, hence the heterogeneity, coupled with right handedness causing external influence to bone turnover. The left is less heterogeneous for the younger group as activity is contributing less to changes in bone chemistry.

Despite the substantial overlap in the 2D scores plots, the influence of phosphate caused a chemical distinction in the left lateral measurements of the middle group, this could be due to the lower rate of bone turnover in the lateral component of the clavicle resulting in higher mineralisation by the time peak bone stability is reached. The influence of carbonate and the proteins amideI and amideIII show a stronger influence on the younger group of the right side, a pattern not apparent when the data was not separated by measurement location. This is likely due to increased bone turnover in the developing skeleton which is still developing its collagen scaffold and the increased carbonate is likely from the need of the mineral component to increase solubility to allow for optimum bone remodelling, an activity for which the addition of carbonate to hydroxyapatite is crucial [18, 19].

Comparison of the medial versus lateral ratios shows a consistently higher ratio in the medial measurements on both sides, for every age group. This aligns with the medial part of the clavicle undergoing more bone turnover, and therefore more chemical changes than the lateral component [37].

### Analysis by age and side: Lateral vs. Middle vs. Medial

When all three measurement areas were considered and compared within each age group, the pattern of increased mineralisation with age and decreasing protein maintained, but was less clear (Fig 5). This analysis found the left and right measurements from the oldest group are the most homogeneous in each measurement area, with the exception of the left medial measurements which align more with the distribution found in the middle group medial measurements (Fig 5B, 5F and 5J). This pattern is likely a result of the decrease in bone turnover with age resulting in more homogeneous bone chemistry. However, the dominant right handedness and higher bone turnover in the medial aspect of the clavicle, established in earlier results (Fig 4), caused slower degeneration to the left medial clavicle than in the right of the older group.

Carbonate did not substantially contribute to distributions in these analyses and there was too much overlap in its presence to determine potential patterns. Phosphate changes, however, contributed to all distributions except the medial LD2. This is potentially linked to the lack of homogeneity in the older group’s left medial measurement. If mineralisation increases with aging and a notable phosphate presence was not detected in the cause of LD2 distribution, this could be why.

Across all analyses where data were split by side, the phosphate intensity was highest in the oldest group, but notably the presence of phosphate was always found to be higher in the right than the left. This indicates higher mineralisation in the dominant side which is further evidence to support the hypothesis that handedness causes changes in bone mineral chemistry, as well as aging. This increase in phosphate is likely a response to increased biomechanical stress across the bone from activity, particularly for these individuals who engaged in very manual work aboard the *Mary Rose*.

A pattern was also found across analyses in the mineral ratio. Though not considerable, these results indicate that P:C is potentially always higher in the middle group in every analysis. This could be supporting the chemical stability that is assumed occurs in the adult skeleton at ~30years [18].

### Analysis by age and side: Measurement location along length of clavicle

The final analyses investigated patterns in bone chemistry changes across the length of the clavicle (Fig 6 and S1 Fig). The analyses of the left and right in the younger group were different. The left had a gradual split in chemistry from lateral to medial as a result of changes in carbonate (~1070cm^-1^) though the intensity of carbonate overlapped too much to identify a pattern. The right side showed alternating chemistry between most consecutive measurements that generally increased from lateral to medial. This could be because of the effect of muscle attachment location, but this is outside the scope of this work. Any changes along the younger right clavicle length were likely caused by changes in phosphate (~960cm^-1^), but the intensities suggested a relatively higher intensity of phosphate in the medial section of the clavicle and lowest in the lateral section. The increase from left to right is a result of a disordered increase in phosphate from medial to lateral, with a higher mineralisation in the middle of the clavicle. This supports previous findings that the middle portion of the clavicle is the most mineralised, and that is why it is the most prone to fracture [30, 38, 39].

Analyses of the middle group were also different between left and right. The left clavicle appeared to show more chemical similarity at the ends than the middle, which was attributed to changes in carbonate. However, whilst it was the biggest chemical change occurring along the length of the left clavicle, the change was not detectable in the averages and was therefore minimal. The right clavicle showed more heterogeneity at each measurement location, but no pattern could be detected. However, the loadings indicated that there was a substantial change in both mineral components, but these did not influence the scores plots sufficiently to detect a trend.

Analysis of the oldest group showed more homogeneity, with the exception of two medial measurements of left clavicles as well as the primary measurement of the right clavicles. In the left this was likely due to the relatively higher mineralisation in the non-dominant side and an increase in mineral with aging. In the right clavicles the primary measurement likely showed higher levels of phosphate as it was taken at the most lateral point, closest to the joint where the most signs of chemical changes with aging would appear when considering handedness. This potentially reflects the beginnings of age-related changes in bone that could be a precursor to a condition like osteoarthritis (OA). OA at the acromioclavicular joint (where the lateral end of the clavicle articulates with the shoulder-blade) is incredibly common, with 89% of individuals over the age of 40 showing OA AC joint pathology and 75% in asymptomatic individuals [40]. The loadings indicate that these distributions were a result of phosphate changes in both clavicles, and amideI in the right. Though as the intensities indicated overlap between the age groups, these could not be attributed to a pattern of chemical change along the clavicle length.

Ratios did not provide a clear pattern of protein and mineral chemical changes along the clavicle length (S1 Fig), but did demonstrate that the degree of irregularity in P:A warrants further investigation as these extreme fluctuations could provide insight into the influence of muscle attachment on bone chemistry.

## Conclusions

This unique study demonstrates that protein has been well preserved in the skeletal remains of the *Mary Rose* sailors by the presence of strong protein peaks at 1450cm^-1^. This allowed analyses of bone collagen and bone mineral. Mineral was found to increase with age in the clavicles as protein decreased, regardless of analyses. Overall, the mineral increase with age was more substantial than the protein decrease. This was influenced by handedness, which was assumed to be the right hand given the nature of the work aboard the ship and the time period in which these men lived. Though patterns were the same when the clavicles were analysed by side, the right side showed more defined changes with aging than the left. This is potentially a result of the increase in bone turnover from external biomechanical stresses and was found even in the younger individuals whose skeletons were still developing. The patterns of bone chemistry changes with aging were therefore concluded to be affected by handedness in the Mary Rose clavicles. To better understand these distributions, more research is needed on the clavicles generally to help identify the causes for these patterns. Additional work using different spectroscopic techniques in conjunction with imaging practices such as Computed Tomography Osteoabsorptiometry (CTOAM) and Nuclear Magnetic Resonance (NMR) may yield more information about these significant historical samples. These techniques have shown that they can distinguish novel macro- and micro- anatomical information about bone composition, and functional abilities in health and disease [41, 42].

This study also identifies that the use of Raman spectroscopy can provide detailed chemical analyses of bone mineral that differs from the abilities of current clinical technologies and research which predominantly use macroscopic analysis to assess bone mineral. Interpreting the patterns in this study allows us to learn more about the livelihoods of historical individuals, the effect their lifestyle had on their body, and how this technique and these results could be used to improve modern clinical understanding of bone chemistry changes with age in the clavicle, with potential future work examining modern samples.

## Supporting information

S1 Fig Ratio plots for the measurements along clavicle length lateral to medial analysed by age and side. (TIF)

S1 Table Clavicle specimen and data collection record. (DOCX)

S2 Table Mineral crystal maturity: Full width of the phosphate peak at half the height, ranked. This demonstrates that crystal maturity differences were negligible across analyses.
(DOCX)
