# Supplementary figures and images for: Shining light on the Mary Rose: Identifying chemical differences in human aging and handedness in the clavicles of sailors using Raman spectroscopy
Source: PLoS One. 2024 Oct 30;19(10):e0311717. doi: 10.1371/journal.pone.0311717 (PMC11524499; doi:10.1371/journal.pone.0311717)

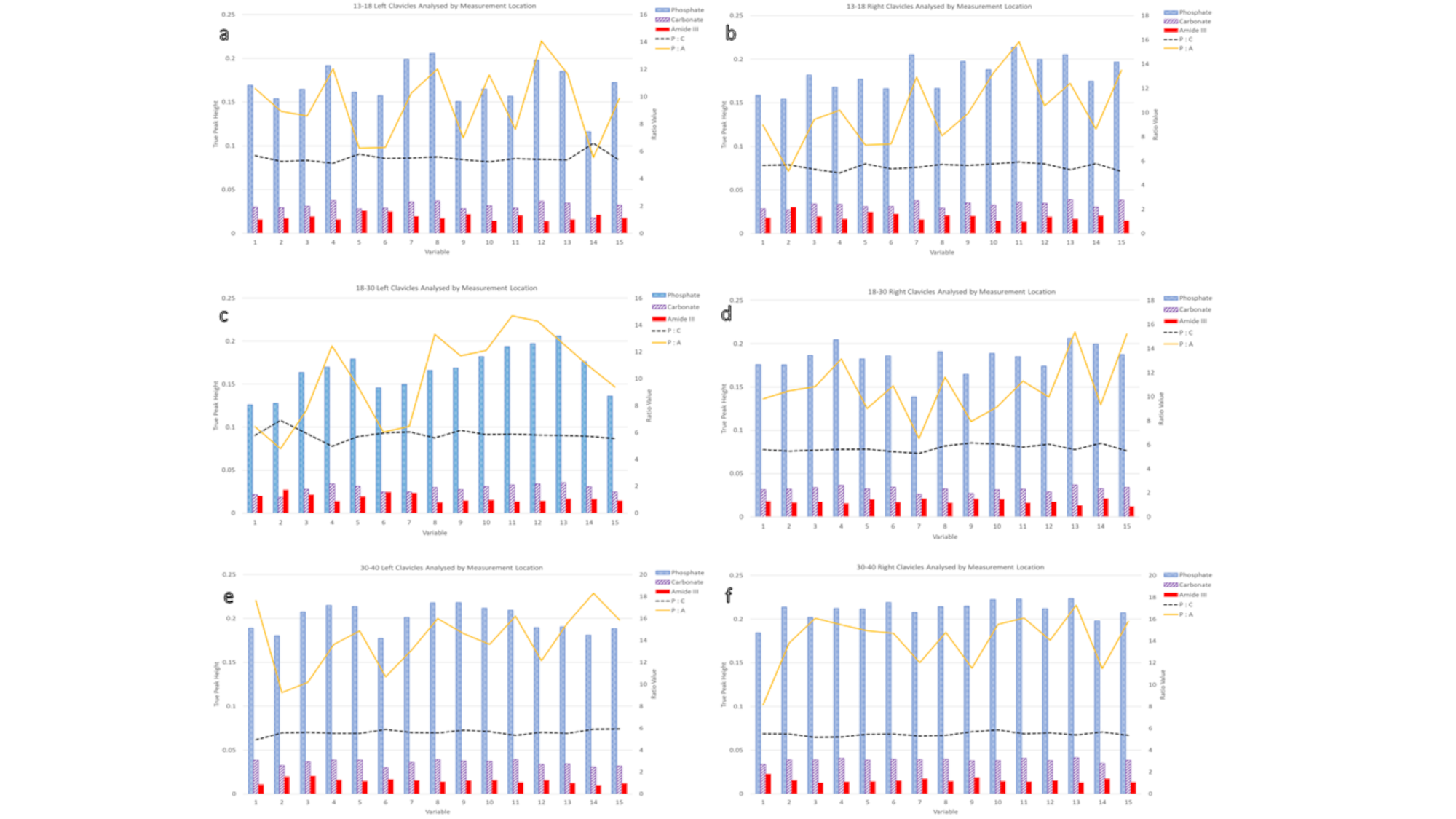

Supplement: S1 Fig — (TIF) [file pone.0311717.s001.tif]
