# Supplementary material for: Shining light on the Mary Rose: Identifying chemical differences in human aging and handedness in the clavicles of sailors using Raman spectroscopy
Source: PLoS One. 2024 Oct 30;19(10):e0311717. doi: 10.1371/journal.pone.0311717 (PMC11524499; doi:10.1371/journal.pone.0311717)
Supplement: S1 Table — (DOCX) [file pone.0311717.s002.docx]

| Skeleton Number | Estimated Age | Clavicle | Area of Measurement | Number of Spectra |
| --- | --- | --- | --- | --- |
| FCS#8 | 18-30 | L | Ant Medial | 5 |
|  |  |  | Anterior Middle | 5 |
|  |  |  | Anterior Lateral | 5 |
|  |  | R | Ant Medial | 5 |
|  |  |  | Anterior Middle | 5 |
|  |  |  | Anterior Lateral | 5 |
| FCS#15 | 13-18 | L | Ant Medial | 5 |
|  |  |  | Anterior Middle | 5 |
|  |  |  | Anterior Lateral | 5 |
|  |  | R | Ant Medial | 5 |
|  |  |  | Anterior Middle | 5 |
|  |  |  | Anterior Lateral | 5 |
| FCS#16 | 30-40 | L | Ant Medial | 5 |
|  |  |  | Anterior Middle | 5 |
|  |  |  | Anterior Lateral | 5 |
|  |  | R | Ant Medial | 5 |
|  |  |  | Anterior Middle | 5 |
|  |  |  | Anterior Lateral | 5 |
| FCS#27 | 30-40 | L | Ant Medial | 5 |
|  |  |  | Anterior Middle | 5 |
|  |  |  | Anterior Lateral | 5 |
|  |  | R | Ant Medial | 5 |
|  |  |  | Anterior Middle | 5 |
|  |  |  | Anterior Lateral | 5 |
| FCS#28 | 13-18 | L | Ant Medial | 5 |
|  |  |  | Anterior Middle | 5 |
|  |  |  | Anterior Lateral | 5 |
|  |  | R | Ant Medial |  |
|  |  |  | Anterior Middle |  |
|  |  |  | Anterior Lateral |  |
| FCS#37 | 18-30 | L | Ant Medial | 5 |
|  |  |  | Anterior Middle | 5 |
|  |  |  | Anterior Lateral | 5 |
|  |  | R | Ant Medial | 6 |
|  |  |  | Anterior Middle | 6 |
|  |  |  | Anterior Lateral | 6 |
| FCS#56 | 13-18 | L | Ant Medial | 5 |
|  |  |  | Anterior Middle | 5 |
|  |  |  | Anterior Lateral | 5 |
|  |  | R | Ant Medial | 5 |
|  |  |  | Anterior Middle | 5 |
|  |  |  | Anterior Lateral | 5 |
| FCS#64 | 18-30 | L | Ant Medial | 5 |
|  |  |  | Anterior Middle | 5 |
|  |  |  | Anterior Lateral | 5 |
|  |  | R | Ant Medial | 5 |
|  |  |  | Anterior Middle | 5 |
|  |  |  | Anterior Lateral | 5 |

| FCS#70 | 18-30 | L | Ant Medial |  |
| --- | --- | --- | --- | --- |
|  |  |  | Anterior Middle |  |
|  |  |  | Anterior Lateral |  |
|  |  | R | Ant Medial | 5 |
|  |  |  | Anterior Middle | 5 |
|  |  |  | Anterior Lateral | 5 |
| FCS#74 | 18-30 | L | Ant Medial | 5 |
|  |  |  | Anterior Middle | 5 |
|  |  |  | Anterior Lateral | 5 |
|  |  | R | Ant Medial | 5 |
|  |  |  | Anterior Middle | 5 |
|  |  |  | Anterior Lateral | 5 |
| FCS#80 | 18-30 | L | Ant Medial | 5 |
|  |  |  | Anterior Middle | 5 |
|  |  |  | Anterior Lateral | 5 |
|  |  | R | Ant Medial | 6 |
|  |  |  | Anterior Middle | 6 |
|  |  |  | Anterior Lateral | 6 |
| FCS#84 | 30-40 | L | Ant Medial | 5 |
|  |  |  | Anterior Middle | 5 |
|  |  |  | Anterior Lateral | 5 |
|  |  | R | Ant Medial | 5 |
|  |  |  | Anterior Middle | 5 |
|  |  |  | Anterior Lateral | 5 |

**supplementarytable1**. Clavicle specimen and data collection record
