# Supplementary material for: Shining light on the Mary Rose: Identifying chemical differences in human aging and handedness in the clavicles of sailors using Raman spectroscopy
Source: PLoS One. 2024 Oct 30;19(10):e0311717. doi: 10.1371/journal.pone.0311717 (PMC11524499; doi:10.1371/journal.pone.0311717)
Supplement: S2 Table — This demonstrates that crystal maturity differences were negligible across analyses. (DOCX) [file pone.0311717.s003.docx]

| **Table of Mineral Crystal Maturity in Each Analysis of the Mary Rose Clavicles** | | | | | |
| --- | --- | --- | --- | --- | --- |
|  | **Sample** | **FWHH** | **Centre** | **Rank** | **Notes** |
| Analysed by Age | 13-18 | 16.46828 | 962.60156 | **2** |  |
|  | 18-30 | 16.63524 | 961.46094 | **3** | least mature |
|  | 30-40 | 16.37213 | 961.46094 | **1** | most mature |
| Analysed by Age & Side | 13-18 Left Clavicle | 16.56998 | 962.60156 | **5** |  |
|  | 13-18 Right Clavicle | 16.27931 | 961.46094 | **1** | most mature |
|  | 18-30 Left Clavicle | 16.8012 | 961.46094 | **6** | least mature |
|  | 18-30 Right Clavicle | 16.536 | 961.46094 | **4** |  |
|  | 30-40 Left Clavicle | 16.4507 | 961.46094 | **3** |  |
|  | 30-40 Right Clavicle | 16.30182 | 961.46094 | **2** |  |
| Analysed by Age: Medial vs Lateral | Left 13-18 Lateral | 16.68529 | 962.60156 | **8** |  |
|  | Left 13-18 Medial | 16.69735 | 962.60156 | **10** |  |
|  | Left 18-30 Lateral | 17.06561 | 961.46094 | **12** | least mature |
|  | Left 18-30 Medial | 16.60704 | 961.46094 | **7** |  |
|  | Left 30-40 Lateral | 16.4689 | 961.46094 | **5** |  |
|  | Left 30-40 Medial | 16.59037 | 961.46094 | **6** |  |
|  | Right 13-18 Lateral | 16.69064 | 961.46094 | **9** |  |
|  | Right 13-18 Medial | 16.01313 | 961.46094 | **1** | most mature |
|  | Right 18-30 Lateral | 16.71689 | 961.46094 | **11** |  |
|  | Right 18-30 Medial | 16.46581 | 961.46094 | **4** |  |
|  | Right 30-40 Lateral | 16.31762 | 961.46094 | **3** |  |
|  | Right 30-40 Medial | 16.18068 | 961.46094 | **2** |  |
| Analysed by Age: Left vs Right Medial vs. Middle vs. Lateral | 13-18 Left Lateral | 16.68529 | 962.60156 | **13** |  |
|  | 13-18 Right Lateral | 16.69064 | 961.46094 | **14** |  |
|  | 18-30 Left Lateral | 17.06561 | 961.46094 | **18** | least mature |
|  | 18-30 Right Lateral | 16.71689 | 961.46094 | **16** |  |
|  | 30-40 Left Lateral | 16.4689 | 961.46094 | **9** |  |
|  | 30-40 Right Lateral | 16.31762 | 961.46094 | **3** |  |
|  | 13-18 Left Middle | 16.38135 | 962.60156 | **5** |  |
|  | 13-18 Right Middle | 16.33091 | 961.46094 | **4** |  |
|  | 18-30 Left Middle | 16.79833 | 962.60156 | **17** |  |
|  | 18-30 Right Middle | 16.47649 | 961.46094 | **10** |  |
|  | 30-40 Left Middle | 16.38415 | 961.46094 | **6** |  |
|  | 30-40 Right Middle | 16.42023 | 961.46094 | **7** |  |
|  | 13-18 Left Medial | 16.69735 | 962.60156 | **15** |  |
|  | 13-18 Right Medial | 16.27931 | 961.46094 | **2** |  |
|  | 18-30 Left Medial | 16.60704 | 961.46094 | **12** |  |
|  | 18-30 Right Medial | 16.46581 | 961.46094 | **8** |  |
|  | 30-40 Left Medial | 16.59037 | 961.46094 | **11** |  |
|  | 30-40 Right Medial | 16.18068 | 961.46094 | **1** | most mature |

**supplementarytable2**. Mineral crystal maturity: full width of the phosphate peak at half the height, ranked. This demonstrates that crystal maturity differences were negligible across analyses.

|  | **Sample** | **FWHH** | **Centre** | **Rank** | **Notes** |
| --- | --- | --- | --- | --- | --- |
| Analysed by Measurement: Left 13-18 only | 13-18 Left Clavicle_1 | 16.81889 | 961.46094 | **10** |  |
|  | 13-18 Left Clavicle_2 | 16.93955 | 962.60156 | **14** |  |
|  | 13-18 Left Clavicle_3 | 16.82093 | 962.60156 | **11** |  |
|  | 13-18 Left Clavicle_4 | 16.36779 | 961.46094 | **5** |  |
|  | 13-18 Left Clavicle_5 | 16.56982 | 962.60156 | **9** |  |
|  | 13-18 Left Clavicle_6 | 16.90319 | 962.60156 | **13** |  |
|  | 13-18 Left Clavicle_7 | 16.17894 | 961.46094 | **2** |  |
|  | 13-18 Left Clavicle_8 | 16.07039 | 962.60156 | **1** | most mature |
|  | 13-18 Left Clavicle_9 | 16.25168 | 962.60156 | **3** |  |
|  | 13-18 Left Clavicle_10 | 16.53577 | 962.60156 | **8** |  |
|  | 13-18 Left Clavicle_11 | 16.43135 | 962.60156 | **7** |  |
|  | 13-18 Left Clavicle_12 | 16.3593 | 962.60156 | **4** |  |
|  | 13-18 Left Clavicle_13 | 16.40297 | 961.46094 | **6** |  |
|  | 13-18 Left Clavicle_14 | 17.70516 | 962.60156 | **15** | least mature |
|  | 13-18 Left Clavicle_15 | 16.85415 | 962.60156 | **12** |  |
| Analysed by Measurement: Right 13-18 only | 13-18 Right Clavicle 1 | 16.49988 | 962.60156 | **10** |  |
|  | 13-18 Right Clavicle 2 | 16.67209 | 961.46094 | **13** |  |
|  | 13-18 Right Clavicle 3 | 16.93234 | 962.60156 | **15** | least mature |
|  | 13-18 Right Clavicle 4 | 16.49186 | 962.60156 | **9** |  |
|  | 13-18 Right Clavicle 5 | 16.61024 | 962.60156 | **12** |  |
|  | 13-18 Right Clavicle 6 | 16.74867 | 962.60156 | **14** |  |
|  | 13-18 Right Clavicle 7 | 16.092 | 961.46094 | **4** |  |
|  | 13-18 Right Clavicle 8 | 16.57853 | 962.60156 | **11** |  |
|  | 13-18 Right Clavicle 9 | 16.08066 | 961.46094 | **3** |  |
|  | 13-18 Right Clavicle 10 | 16.31041 | 961.46094 | **7** |  |
|  | 13-18 Right Clavicle 11 | 15.70799 | 961.46094 | **2** |  |
|  | 13-18 Right Clavicle 12 | 16.12492 | 962.60156 | **5** |  |
|  | 13-18 Right Clavicle 13 | 15.82871 | 961.46094 | **1** | most mature |
|  | 13-18 Right Clavicle 14 | 16.41568 | 961.46094 | **8** |  |
|  | 13-18 Right Clavicle 15 | 16.27636 | 962.60156 | **6** |  |
| Analysed by Measurement: Left 18-30 only | 18-30 Left Clavicle 1 | 17.22311 | 962.60156 | **14** |  |
|  | 18-30 Left Clavicle 2 | 17.65569 | 962.60156 | **15** | least mature |
|  | 18-30 Left Clavicle 3 | 16.99781 | 961.46094 | **12** |  |
|  | 18-30 Left Clavicle 4 | 16.96328 | 961.46094 | **11** |  |
|  | 18-30 Left Clavicle 5 | 16.87724 | 961.46094 | **8** |  |
|  | 18-30 Left Clavicle 6 | 16.92434 | 961.46094 | **10** |  |
|  | 18-30 Left Clavicle 7 | 17.12736 | 962.60156 | **13** |  |
|  | 18-30 Left Clavicle 8 | 16.87107 | 962.60156 | **7** |  |
|  | 18-30 Left Clavicle 9 | 16.86579 | 962.60156 | **6** |  |
|  | 18-30 Left Clavicle 10 | 16.54768 | 962.60156 | **3** |  |
|  | 18-30 Left Clavicle 11 | 16.50878 | 962.60156 | **2** |  |
|  | 18-30 Left Clavicle 12 | 16.57508 | 961.46094 | **4** |  |
|  | 18-30 Left Clavicle 13 | 16.37209 | 961.46094 | **1** | most mature |
|  | 18-30 Left Clavicle 14 | 16.67169 | 962.60156 | **5** |  |
|  | 18-30 Left Clavicle 15 | 16.89675 | 962.60156 | **9** |  |

|  | **Sample** | **FWHH** | **Centre** | **Rank** | **Notes** |
| --- | --- | --- | --- | --- | --- |
| Analysed by Measurement: Right 18-30 only | 18-30 Right Clavicle 1 | 16.82808 | 962.60156 | **14** |  |
|  | 18-30 Right Clavicle 2 | 16.6973 | 961.46094 | **8** |  |
|  | 18-30 Right Clavicle 3 | 16.73147 | 961.46094 | **10** |  |
|  | 18-30 Right Clavicle 4 | 16.7747 | 961.46094 | **12** |  |
|  | 18-30 Right Clavicle 5 | 16.61141 | 962.60156 | **6** |  |
|  | 18-30 Right Clavicle 6 | 16.61442 | 961.46094 | **7** |  |
|  | 18-30 Right Clavicle 7 | 17.05706 | 961.46094 | **15** | least mature |
|  | 18-30 Right Clavicle 8 | 16.40771 | 961.46094 | **4** |  |
|  | 18-30 Right Clavicle 9 | 16.73215 | 962.60156 | **11** |  |
|  | 18-30 Right Clavicle 10 | 16.34114 | 961.46094 | **2** |  |
|  | 18-30 Right Clavicle 11 | 16.4006 | 961.46094 | **3** |  |
|  | 18-30 Right Clavicle 12 | 16.77866 | 961.46094 | **13** |  |
|  | 18-30 Right Clavicle 13 | 16.44097 | 961.46094 | **5** |  |
|  | 18-30 Right Clavicle 14 | 16.29456 | 961.46094 | **1** | most mature |
|  | 18-30 Right Clavicle 15 | 16.71912 | 962.60156 | **9** |  |
| Analysed by Measurement: Left 30-40 only | 30-40 Left Clavicle 1 | 16.63748 | 962.60156 | **11** |  |
|  | 30-40 Left Clavicle 2 | 16.46336 | 961.46094 | **6** |  |
|  | 30-40 Left Clavicle 3 | 16.43767 | 961.46094 | **5** |  |
|  | 30-40 Left Clavicle 4 | 16.49052 | 961.46094 | **8** |  |
|  | 30-40 Left Clavicle 5 | 16.56999 | 961.46094 | **9** |  |
|  | 30-40 Left Clavicle 6 | 16.79045 | 962.60156 | **14** |  |
|  | 30-40 Left Clavicle 7 | 16.33508 | 962.60156 | **3** |  |
|  | 30-40 Left Clavicle 8 | 16.4695 | 961.46094 | **7** |  |
|  | 30-40 Left Clavicle 9 | 16.24514 | 961.46094 | **2** |  |
|  | 30-40 Left Clavicle 10 | 16.12302 | 961.46094 | **1** | most mature |
|  | 30-40 Left Clavicle 11 | 16.59572 | 961.46094 | **10** |  |
|  | 30-40 Left Clavicle 12 | 16.64684 | 961.46094 | **12** |  |
|  | 30-40 Left Clavicle 13 | 17.04313 | 961.46094 | **15** | least mature |
|  | 30-40 Left Clavicle 14 | 16.3581 | 961.46094 | **4** |  |
|  | 30-40 Left Clavicle 15 | 16.72735 | 961.46094 | **13** |  |
| Analysed by Measurement: Right 30-40 only | 30-40 Right Clavicle 1 | 16.55111 | 962.60156 | **14** |  |
|  | 30-40 Right Clavicle 2 | 16.1405 | 961.46094 | **3** |  |
|  | 30-40 Right Clavicle 3 | 16.33565 | 962.60156 | **9** |  |
|  | 30-40 Right Clavicle 4 | 16.29515 | 961.46094 | **7** |  |
|  | 30-40 Right Clavicle 5 | 16.46625 | 961.46094 | **12** |  |
|  | 30-40 Right Clavicle 6 | 16.40122 | 961.46094 | **10** |  |
|  | 30-40 Right Clavicle 7 | 16.59696 | 962.60156 | **15** | least mature |
|  | 30-40 Right Clavicle 8 | 16.46105 | 961.46094 | **11** |  |
|  | 30-40 Right Clavicle 9 | 16.54382 | 961.46094 | **13** |  |
|  | 30-40 Right Clavicle 10 | 16.23064 | 961.46094 | **4** |  |
|  | 30-40 Right Clavicle 11 | 16.27319 | 961.46094 | **5** |  |
|  | 30-40 Right Clavicle 12 | 16.33071 | 961.46094 | **8** |  |
|  | 30-40 Right Clavicle 13 | 16.09125 | 961.46094 | **2** |  |
|  | 30-40 Right Clavicle 14 | 16.00572 | 962.60156 | **1** | most mature |
|  | 30-40 Right Clavicle 15 | 16.27373 | 961.46094 | **6** |  |
